# Prevention of Sudden Death in Hypertrophic Cardiomyopathy

**DOI:** 10.5935/abc.20180101

**Published:** 2018-07

**Authors:** Edmundo Arteaga-Fernández, Murillo de Oliveira Antunes

**Affiliations:** Instituto do Coração do Hospital das Clínicas da Faculdade de Medicina da Universidade de São Paulo (INCOR HC FMUSP), São Paulo, SP - Brazil

**Keywords:** Cardiomyopathy, Hypertrophic, Death, Sudden, Cardiac / prevention & control, Heart Defects, Congenital

Hypertrophic cardiomyopathy (HCM) is the most common congenital disease, and sudden death (SD), its most feared complication, was already mentioned by Donald Teare^1^ in the first description of the disease, being observed in 7 out of 8 patients. SD occurs during daily activities, after exercises and even during sleep; it may affect young athletes, which has a great impact on the media. This has required considerable effort by researchers in defining clinical factors and complementary tests that could be used in the screening of individuals at higher risk that could benefit from implantable cardioverter defibrillator (ICD) and also to prevent SD, since it is caused by tachycardia and ventricular fibrillation.^2^ HCM favors the occurrence of ventricular arrythmias - hypertrophy causes repolarization dispersion; myocyte disarray and increased fibrosis create areas of conduction block and predispose to reentry arrhythmias; and abnormalities in ion fluxes, such as calcium, during repolarization may also trigger arrhythmias. In addition, this complex arrhythmogenic substrate may be modulated by impaired autonomic response, myocardial ischemia and left ventricular outflow tract obstruction.^2-4^ If we consider deaths from cardiovascular causes, in patients with HCM, they account for 0.5%-1.5% deaths a year, which is near to that of the general population.^2^ In HCM patients considered as high risk, SD may reach 2.5% of deaths a year.^5^ However, the accurate identification of these patients for preventive therapy with ICD may be challenging.

Before the guidelines were published,^3^ it was known that manifestations of HCM in children younger than 10 years old with diastolic or systolic dysfunction, SD in first-degree relatives younger than 50 years, nonsustained ventricular tachycardia, syncope and myocardial hypertrophy > 30 mm were factors associated with SD, and the last four fully considered as indications for ICD in the first guideline (2011).^2^

Today, we know that the positive predictive value of each of these factors is low, and there is little evidence suggesting a higher predictive value of any of these factors. However, some authors have considered only one risk factor for indication of ICD.^6^

The two largest multicentric studies grounded in the American guidelines^2^ - one of adults (n = 506, mean age of 42; mean follow-up period of 3.7 years) showed that for primary prevention ICD indication, in 75% of cases, the devices were used in 4%/year, whereas for the secondary prevention, intervention rates were 12%/year in 25% of cases. Therapies were found in 20% of patients and inappropriate shocks in 27%, with 7% of complications.^7^ The other study involved 224 children and adolescents (mean age of 14 years; mean follow-up of 4.3 years). Primary prevention was indicated in 84% of cases and secondary prevention indicated for 16% of cases. Intervention rates were similar to those in adults, with therapies and inappropriate shocks in 19% and 41% of cases, respectively.^8^

The 2014 European Guidelines (ESC) recommended a new sudden-death risk model based on a longitudinal, retrospective, multicenter study risk calculation model (n = 3,675) and seven variables - age, history of SD, syncope, wall thickness, left atrial diameter, left ventricular outflow gradient and nonsustained ventricular tachycardia. In primary prevention, the risk calculation encompasses three SD risk levels at five years - low, moderate and high - for patients older than 16 years.^3,9^ This risk prediction model, validated in Europe^10^ (n = 706) and in South America^11^ (n = 502), was shown to better predict individual risks as compared with that used in North America and Canada societies. However, a study^12^ using the ESC risk calculation model (n = 1,629, age > 16 years) showed that most patients with HCM or with previous ICD were classified as low risk and therefore would remain unprotected from SD. The authors concluded that the primary risk stratification using this model is unreliable for prediction of future SD events.^13^

In this issue of *Arquivos Brasileiros de Cardiologia*, in a cohort study (n = 105), Reis et al.^14^ compared the American and the European guidelines in stratifying SD risk, and concluded that the European model reduces the proportion of patients with indication for ICD.

We can affirm that, despite continuous advances in knowledge,^10,15^ the assessment of SD risk in HCM is limited to a small number of patients (5%) and is still a great challenge. The guidelines have so far included increasing number of risk factors^15^ with low predictive value, and validated for a frequent, but still underdiagnosed disease, characterized by patients with a normal life cycle and free from SD.
